# IL-40 levels in treatment-naive and methotrexate-treated rheumatoid arthritis patients

**DOI:** 10.55730/1300-0144.6013

**Published:** 2025-03-24

**Authors:** Hamdi OĞUZMAN, Mete PEKDİKER

**Affiliations:** 1 Department of Medical Biochemistry, Faculty of Medicine, Hatay Mustafa Kemal University, Hatay, Turkiye; 2 Department of Rheumatology-Internal Medicine, Faculty of Medicine, Hatay Mustafa Kemal University, Hatay, Turkiye

**Keywords:** Interleukin-40, methotrexate, cytokines, transforming growth factor beta-1, interleukin-4, tumor necrosis factor-alpha

## Abstract

**Background/aim:**

Rheumatoid arthritis (RA) is an autoimmune disease that is characterized by persistent inflammation and progressive damage to the joints. In this study, it was aimed to explore the role of interleukin (IL)-40, a newly discovered cytokine, in the pathogenesis of RA. We also aimed to investigate the relationship between IL-40 and other cytokines such as IL-4, transforming growth factor (TGF)-β1 and tumor necrosis factor (TNF)-α.

**Materials and methods:**

This single-center, cross-sectional study included 87 participants divided into three groups: healthy controls (n = 29), newly diagnosed RA patients (n = 29), and RA patients with remission under methotrexate (MTX) monotherapy (n = 29). Serum samples were collected and analyzed for IL-40, IL-4, TGF-β1 and TNF-α levels using ELISA. Disease activity score, presence of autoantibodies and other relevant clinical data were obtained from hospital electronic records.

**Results:**

Elevated IL-40 levels were found in the newly diagnosed RA patients and in those treated with MTX compared to the control group (p < 0.001). Logistic regression analysis confirmed IL-40 as an independent predictor in the newly diagnosed (OR = 1.023, 95% CI: 1.010–1.035, p = 0.002) and RA MTX-treated patients (OR = 1.023, 95% CI: 1.011–1.036, p < 0.001). IL-40 levels remained unchanged in the newly diagnosed RA group compared to RA patients in the MTX-treated group. In the dual-seropositive patients, TGF-β1 was lower in the MTX-treated RA patients compared to the naive patients (p = 0.013) and in the dual-seronegative patients, TNF-α was decreased in the MTX-treated RA patients in comparison to naive patients (p = 0.043).

**Conclusion:**

This study demonstrates that IL-40 levels are elevated in RA patients and highlights its potential role in RA. The fact that IL-40 levels did not change despite the antiinflammatory effects of MTX suggests that IL-40 is involved in immunological pathways that are less responsive to treatment.

## 1. Introduction

Rheumatoid arthritis (RA) is an autoimmune, and multisystemic rheumatologic disease, and it has a chronic, and progressive course. The prevalence of RA is 0.5%–1%, and there is a clear female predominance. Chronic synovitis is the hallmark lesion of RA; bone, and cartilage erosions occur. Joint erosions, and deformities lead to morbidity; extra-articular manifestations lead to an increase in mortality during the course of RA [1].

The precise etiology of RA remains unclear. A combination of genetic predisposition and environmental factors play a role in its development. The pathogenesis of RA involves interactions between the innate and adaptive immune systems, where B cells, T cells, macrophages, and neutrophils work together in a coordinated manner [2]. Cytokines have multiple functions, and regulate the immune system, so they have a pivotal role in the pathogenesis of RA. Tumor necrosis factor (TNF)-α and interleukin (IL)-6 are key effector cytokines, while various other interleukins and cytokines also play roles in inflammation [3]. Therefore, patients with RA who have not responded to traditional synthetic disease-modifying antirheumatic drugs (csDMARDs) may benefit from biologic therapies (BA), which include medications that block cytokines or their receptors, such as anti-TNF, anti-IL-1, and anti-IL-6 agents [4,5].

Interleukin-40 is a recently identified cytokine associated with B cells. It is encoded by the *C17orf99* gene on chromosome 17 and has been shown to be expressed by fetal liver, bone marrow and activated B cells. It has been included in the orphan cytokine classification in studies [6]. Studies have implicated transforming growth factor (TGF)-beta1 and IL-4 in the regulation of IL-40 secretion. In particular, the role of IL-40 together with IL-4 in B cell maturation and humoral immunity is noteworthy [6,7]. Recent studies in RA patients indicate that IL-40 is elevated in the serum and synovial fluid of RA patients [8]. Given the recently developed treatment options for B cells and the cytokines secreted by these cells in RA patients, it has become very important to identify the cytokines involved in B cell activation in RA patients [9].

In this context, it is important to understand the role of IL-40 cytokine in RA patients. Despite novel therapies for rheumatic diseases, unmet treatment needs are still a major problem in daily practice [10], and growing evidence on cytokines is still ongoing. In this research, we aimed to analyze the serum levels of IL-40 in newly diagnosed-DMARD naive RA patients, and its association with disease parameters and MTX treatment..

## 2. Materials and methods

### 2.1. Patients

We conducted this study in a rheumatology department of a tertiary university hospital from January 2024 to December 2024. This study was designed as a cross-sectional analysis and conducted in a single-center. It included three groups: newly diagnosed, treatment-naive RA patients, RA patients in remission under methotrexate (MTX) monotherapy, and a healthy control group.

Group I (Healthy Control Individuals): This group consisted of 29 healthy individuals (21 women, 8 men). All individuals in this group were free of any inflammatory or chronic diseases.

Group II (Newly Diagnosed DMARDs-naive RA patients): This group comprised 29 patients (22 women, 7 men) who were newly diagnosed with RA according to the 2010 American College of Rheumatology (ACR)/European League Against Rheumatism (EULAR) criteria [11]. All patients in this group were treatment-naive, with no prior use of DMARDs, corticosteroids, immunosuppressants, or chemotherapeutic agents.

Group III (RA MTX-treated): This group consisted of 29 patients (22 women, 7 men). All patients achieved clinical remission while on MTX monotherapy, with a disease activity score (DAS)-28 of less than 2.6 [12].

The inclusion criteria for RA patients were as follows: age between 18 and 70 years; diagnosis of RA according to the ACR/EULAR 2010 classification criteria; treatment-naive RA patients for group II; RA patients on MTX monotherapy with a DAS-28 score of less than 2.6 for group III.

Individuals were excluded if they had active infections during the period of serum collection; were diagnosed with another immune-mediated inflammatory disorder or malignancy; had comorbidities including chronic renal disease, chronic heart failure, chronic obstructive pulmonary disease (COPD), or uncontrolled diabetes mellitus (HbA1c >10 g/dL); were pregnant or breastfeeding; had a history of biological DMARD (bDMARD) or targeted synthetic DMARD (tsDMARD) use in group III.

### 2.2. Data collection

Patient demographic data, including age, sex, disease duration, presence of autoantibodies (rheumatoid factor [RF] and anticyclic citrullinated peptide antibody IgG [anti-CCP]), acute phase reactants (erythrocyte sedimentation rate [ESR] and C-reactive protein [CRP]), disease activity scores, and hand-wrist radiographic findings, were collected from the hospital’s electronic medical record system. For the determination of serum cytokine levels, blood was collected in 8 mL anticoagulant-free biochemistry tubes. To obtain serum, blood was centrifuged at 1500 g for 10 min after blood collection. The obtained serum samples were aliquoted and stored at −80°C for ELISA analysis.

The ESR was assessed using 4 mL whole blood samples collected in EDTA tubes and examined with the Vision-C analyser (Shenzhen YHLO Biotech Co., Ltd., Shenzhen, China). Serum CRP levels and RF were quantified using the nephelometric technique (Siemens BNII, Siemens Healthcare Diagnostics Inc., Tarrytown, NY, USA). Anti-CCP was quantified by the immunoassay technique (ADVIA Centaur XPT, Siemens Healthcare Diagnostics Inc., Tarrytown, NY, USA). RF levels above 14 IU/mL were considered positive, while anti-CCP levels above 5 U/mL were considered positive.

### 2.3. Outcome measures

Radiographic evaluation of peripheral joints was performed using the Modified Sharp Score (MSS) system. Bone erosions were defined as disruptions of the bone cortex, as per EULAR criteria [13]. Radiological assessment by a rheumatologist who has over ten years of experience. To assess disease severity, the DAS-28 scoring system was used [12].

### 2.4. ELISA parameters analysis

Serum samples were analyzed for IL-40, IL-4, TGF-β1, and TNF-α utilizing commercially available kits in accordance with the manufacturer’s procedure (Thermo Fisher Scientific Multiscan Go, Finland).

The performance specifications of the kits are as follows. All kits had intra-assay and inter-assay coefficients of variation ≤10%.

IL-40 ELISA kit (Human) (Fine Test, Catalog No: EH5091) The analytical range is 31.25 to 2000 pg/mL, with a sensitivity of 18.75 pg/mL.

IL-4 ELISA kit (Human) (Elabscience, Cat. No: E-EL-H0101 The analytical range is 31.25–2000 pg/mL and the sensitivity was 18.75 pg/mL.

TGF-β1 ELISA kit (Human) (Elabscience, Cat. No: E EL-H0110 The analytical range is 31.25–2000 pg/mL and the sensitivity was 18.75 pg/mL.

TNF-α ELISA kit (Human) (Elabscience, Cat. No: E-EL-H0109): The analytical range is 7.81–500 pg/mL and the sensitivity was 4.69 pg/mL.

### 2.5. Sample size and power analysis

A sample size calculation was performed using an expected effect size (f) of 0.40, a type I error rate (α) of 0.05, and a statistical power (1-β) of 0.80. This yielded a minimum sample size of 66 people. A power study was conducted with G*Power version 3.1.9.2 software [14].

### 2.6. Statistical analysis

Data analysis was conducted using SPSS Statistics, version 26.0 (SPSS Inc., Chicago, IL). The Shapiro-Wilk test was used to evaluate the normality of the data. Normally distributed data were expressed as mean ± standard deviation, while nonnormally distributed variables were reported as median (interquartile range). Categorical variables were analyzed using the chi-square test. For continuous variables with a normal distribution, one-way ANOVA was applied, followed by post-hoc Tukey or Tamhane’s T2 tests when necessary. The Kruskal-Wallis test, accompanied by the Bonferroni post-hoc multiple comparison test, was employed for continuous variables that did not exhibit a normal distribution. Relationships between variables were evaluated using the Pearson correlation test for normally distributed data and the Spearman correlation test for nonnormally distributed data. A multinominal logistic regression analysis was performed to assess the relationship between IL-40 levels and the disease groups (reference category = control group), adjusting for both age and gender as possible confounding variables. The results are presented as odds ratios (OR) (95% confidence intervals). P values < 0.05 were considered statistically significant.

## 3. Results

In the study, 3 groups consisting of 29 individuals were investigated. The average age of the individuals showed a significant difference between the groups (p = 0.001). DAS-28 was significantly increased in newly diagnosed RA patients in comparison with the RA MTX-treated group, indicating high disease activity in untreated individuals (p < 0.001). The DAS-28 score was significantly reduced in the RA MTX-treated group, demonstrating the efficacy of MTX therapy in reducing disease activity.

Interleukin-40 levels were significantly increased in both the newly diagnosed RA group and the RA MTX-treated group in comparison to the control group (p < 0.001). Nonetheless, no significant difference was observed between the newly diagnosed RA group and the RA MTX-treated group (p = 1.000), indicating that IL-40 levels remain elevated after MTX therapy. Despite elevated IL-4 levels in the RA groups (the newly diagnosed RA group and the RA MTX-treated group) compared to the control group, the changes were not statistically significant (p = 0.228). TNF-α levels significantly elevated in both RA groups (the newly diagnosed RA group and the RA MTX-treated group) relative to the control group (p < 0.001). TGF-β1 levels were significantly elevated in the newly diagnosed RA group compared to the control group (p = 0.001). Despite a tendency for elevated TGF-β1 levels in the newly diagnosed RA group compared to the RA MTX-treated group, this difference was not statistically significant (p = 0.121) (Table 1). The newly diagnosed RA and RA MTX-treated groups were more likely to have elevated IL-40 levels than healthy subjects, independent of age and gender in the logistic regression analysis (OR = 1.023, 95% CI: 1.010–1.035, p = 0.002; OR = 1.023, 95% CI: 1.011–1.036, p < 0.001, respectively).

The comparison of cytokine levels between the dual-seropositive (RF ≥ 14 IU/mL and anti-CCP ≥ 5 IU/mL and dual-seronegative (RF < 14 IU/mL and anti-CCP < 14 IU/mL) groups of newly diagnosed RA patients is shown in Table 2. IL-4 levels showed statistically significant changes, but other cytokines did not differ considerably between groups. IL-4 concentrations were markedly increased in the dual-seropositive group (14.6 pg/mL ± 7) relative to the dual-seronegative group (7 pg/mL ± 2.4) (p = 0.004). The average TGF-β1 levels were elevated in the dual-seropositive group (1491.6 pg/mL ± 674.8) compared to the dual-seronegative group (1020.5 pg/mL ± 545.4). This trend indicates increased TGF-β1 levels in dual-positive people; nevertheless, the difference was not statistically significant (p = 0.087).

**Figure 1:**
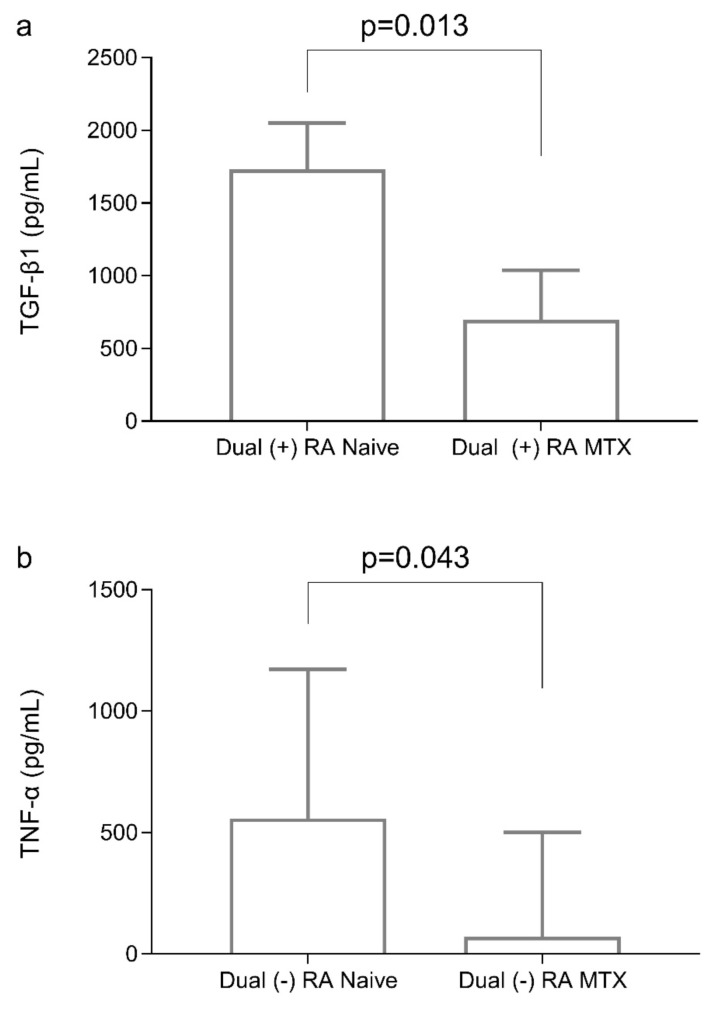
Comparison of TGF-β1 and TNF-α levels in RA patients based on treatment status and serological profile. a. Median TGF-β1 levels were significantly higher in the dual-seropositive treatment-naive RA patients compared to the dual-seropositive RA patients in remission under MTX therapy (p = 0.013). b. Median TNF-α levels were significantly elevated in the dual-seronegative treatment-naive RA patients compared to the dual-seronegative RA patients in remission under MTX therapy (p = 0.043). Dual-seropositive: RF ≥ 14 IU/mL and anti-CCP ≥ 5 U/mL and dual-seronegative: RF < 14 IU/mL and anti-CCP < 5 IU/mL. MTX: Methotrexate, RA: Rheumatoid arthritis TGF-β1: Transforming Growth Factor Beta 1, TNF-α: Tumor Necrosis Factor Alpha.

Transforming growth factor-β1 levels in the dual-seropositive RA patients were elevated in the naive group (1732.7 pg/mL [1080.9–2051.8]) compared to the RA MTX-treated group (699.1 pg/mL [610.1–946.9]) (p = 0.013) (Figure 1a). TNF-α levels in the dual-seronegative RA patients were significantly higher in the naive group (558.1 pg/mL [192.8–819.4]) than in the RA MTX-treated group (71.9 pg/mL [52.3–452.7)] (p = 0.043) (Figure 1b).

In the newly diagnosed RA patients, IL-4 levels showed a significant positive association with RF titer (r = 0.497, p = 0.008) and anti-CCP titer (r = 0.572, p = 0.001). A significant positive association was also detected between TNF-α and TGF-β1 levels (r = 0.423, p = 0.025). No statistically significant correlation was found between IL-40 and any of the other measures.

## 4. Discussion

This study investigated serum IL-40 levels in both drug-naive and those receiving MTX treatment RA patients, and compared them to healthy controls. Our results indicate that IL-40 levels were significantly elevated in RA patients, irrespective of MTX treatment. The consistent elevation of IL-40, despite MTX therapy, suggests that IL-40 may participate in immunological processes that are less responsive to conventional RA treatments. This study also explores the interplay between IL-40 and other cytokines, including TGF-β1, IL-4, and TNF-α, offering insights into the complex cytokine networks that characterize RA and contribute to variations in disease activity and therapeutic response.

There was a significant increase in the levels of IL-40 in both the drug-naive RA and the MTX-treated patients with RA when compared to the healthy controls (p < 0.001). Furthermore, logistic regression analysis showed that IL-40 levels were significantly related to both the newly diagnosed RA patients and the MTX-treated RA patients, independent of age and gender. Additionally, to IL-40, the levels of TGF-β1 were also significantly increased in the RA patients compared to the healthy subjects (p < 0.001). IL-4 levels were also significantly elevated in the newly diagnosed seropositive RA patients (p = 0.004). In one study, TGF-β1 and IL-4 were shown to play an important role in the regulation of IL-40, thus contributing to B-cell activation and autoantibody production [6]. Although TGF-β1 is normally immunosuppressive [15], it paradoxically facilitates autoantibody formation in RA and may work with IL-40 to enhance RF and anti-CCP production [16,17]. Similarly, IL-4 facilitates B-cell differentiation and antibody class switching, thereby enhancing autoantibody synthesis [18]. Our findings align with previous researches linking IL-40 elevation to B-cell activity and autoantibody synthesis in RA [8]. Notably, IL-4 levels showed a significant positive correlation with RF titer (r = 0.497, p = 0.008) and anti-CCP titer (r = 0.572, p = 0.001) in our study, further supporting its role in autoantibody production and humoral immune dysregulation in RA. These results may indicate that in RA patients, IL-40, along with TGF-β1 and IL-4, may be involved in B-cell activation and the synthesis of autoantibodies. However, in our study, we found no significant correlation between IL-40 and RF or anti-CCP in the newly diagnosed RA patients. In contrast, Navrátilová et al. reported elevated IL-40 levels in RF and anti-CCP-positive patients. Their study also demonstrated a strong correlation between IL-40 and autoantibodies [8]. Several factors may explain this discrepancy. First, differences in sample size could play a role, as prior studies included larger sample sizes, potentially increasing the power to detect associations. Additionally, IL-40 is secreted by multiple immune cell types, including neutrophils [19], which may contribute to its persistence. Finally, disease stage may also be a factor, as previous research suggests that IL-40 correlations with RF/anti-CCP may be more pronounced in early RA, whereas its levels in chronic RA may be stabilized by long-term immune dysregulation. Future studies with larger, stratified cohorts are needed to investigate IL-40 and autoantibodies in RA patients.

Another important finding of this study is that there was no difference in serum IL-40 levels between the newly diagnosed RA patients and those treated with MTX. Similarly, a study of individuals with active RA found no difference in IL-40 levels between patients treated with and without MTX [19]. MTX specifically inhibits classical inflammation cytokines like TNF-α and IL-6 [20]. It has been shown that IL-40 levels in RA patients are not affected by TNF inhibitor therapy such as adalimumab, whereas IL-40 levels are decreased by B cell depletion therapy such as rituximab [8]. The observation that MTX does not alter IL-40 levels in our study may be due to the fact that the primary effects of the treatment are not directly related to B cells or various factors that primarily regulate IL-40. However, Navrátilová et al. demonstrated a significant decrease in IL-40 levels after 3 months of conventional RA treatment (MTX and other DMARDs) in active RA patients [21]. These confusing findings may also be caused by the highly variable cytokine profiles observed in RA patients. It is known that cytokine profiles in early RA can differ significantly from those in long-term disease [22]. The study by Navrátilová et al. included patients with early RA who had never been treated for the disease [21]. In contrast, our study included patients with both early and more chronic RA, as well as people with different levels of inflammation at baseline. Navrátilová et al. reviewed the results of two concurrent studies and found that IL-40 levels were approximately twofold lower in patients with newly diagnosed erosive RA compared to those with long-standing RA [8,21]. Their findings support the notion that IL-40 may stabilize in patients with established or chronic RA. In addition, the association between IL-40 levels and genetic variations may differ between genotypes in terms of treatment response. Notably, analysis of the rs2004339 variant showed significantly higher IL-40 levels compared to individuals with a different genotype. In this study, although IL-40 levels differed between genotypes in treated and newly diagnosed patients, the authors noted that these differences were not statistically significant, possibly because of the small sample size [23]. This indicates that genetic variations may have a treatment-dependent effect on IL-40 levels. A study showed that RF and anti-CCP positivity rates in the Turkish population differed from those in other populations, probably due to genetic variations that influence RA development [24]. This finding may suggest that IL-40 levels may also vary according to genetic and demographic factors. The consistent IL-40 levels in both drug-naive and MTX-treated patients in our study may indicate that IL-40 may function differently depending on the mechanism of action of the treatment, disease stage, inflammatory milieu, and genetic factors.

We also investigated the relation of TNF-α with IL-40 and MTX therapy. TNF-α is an important pro-inflammatory cytokine involved in the pathogenesis of RA, particularly in seronegative patients where the inflammatory response tends to be driven more by innate immune cells than by autoantibodies [25]. While high levels of TNF-α were observed in the RA patients, a significant decrease in TNF-α levels was observed in the MTX-treated seronegative RA patients compared to the newly diagnosed seronegative patients. This corresponds with studies indicating that TNF-α inhibitors are especially useful in managing inflammation in seronegative RA patients, reinforcing the notion that TNF-α plays a more pivotal role in disease activity for these individuals than autoantibody-mediated mechanisms in seropositive RA.

### 4.1. Limitations

This study was conducted in a single center, which may introduce location-specific biases. The inclusion of both newly diagnosed and chronic RA patients potentially limits the generalizability of our findings. Moreover, although we discuss the potential impact of genetic variability on IL-40 expression, our study did not include genetic profiling, limiting our ability to evaluate this relationship directly. Lastly, a key limitation of our study is its cross-sectional design, which restricts our ability to track changes in cytokine levels over time. Longitudinal studies are needed to assess whether IL-40 levels fluctuate with disease activity or respond dynamically to treatment. Our findings suggest IL-40 may be a persistent marker of inflammation, but future research should examine its trajectory in RA patients at different disease stages.

### 4.2. Conclusion

This study highlights the potential role of IL-40 in RA by demonstrating its elevated levels in RA patients. There was no change in IL-40 levels with MTX, an antiinflammatory drug. This suggesting IL-40 involvement in immunological pathways that are less responsive to MTX treatment. Furthermore, the results obtained for IL-40, TGF-β1 and IL-4 highlight the potential role of these cytokines in activating B cells. It is suggests that future research should investigate the long-term changes in IL-40 levels and the relationship of IL-40-directed therapies with B-cells. Additionally, genetic variations influencing IL-40 expression should be further investigated to elucidate its role in RA pathogenesis and treatment processes.

## Figures and Tables

**Table 1:** Comparison of clinical and biochemical markers between the control group, naive RA patients, and RA patients receiving MTX treatment.

Parameter	Control (I) (n = 29)	Newly Diagnosed RA (II) (n = 29)	RA MTX-Treated (III) (n = 29)	p-value	I–II	I–III	II–III
Sex (F/M)	21/8	22/7	22/7	0.850*			
Age (year)	38.4 ± 12.1	50.6 ± 14.8	49.8 ± 9.1	0.001**	0.002	0.004	0.961
Disease Duration (month)		12 (6–24)	24 (12–30)	0.002^t^			
DAS-28		4.14 ± 0.83	1.54±0.28	<0.001**			
ESR (mm/h)	9.5 (6–16.5)	29 (17–49)	16 (5–26)	<0.001	<0.001	0.538	0.008
CRP (mg/L)	3 (3–3)	18 (7–26)	3 (1–4)	<0.001	<0.001	1	<0.001
RF (IU/mL)	0 (0–0)	37 (0–80)	25 (0–75.5)	0.001	0.002	0.003	1
anti-CCP (U/mL)	0 (0–0)	7 (0–201)	18 (0–154)	0.055			
IL-40 (pg/mL)	114.85 (64.1–144.8)	240.45 (198.55–388.25)	294.4 (232.8–367.1)	<0.001	<0.001	<0.001	1
IL-4 (pg/mL)	6.55 (5.3–11.4)	8.55 (6.2–13.35)	9 (6.1–11)	0.228			
TGF-β1 (pg/mL)	617.85 (501.8–798.5)	1114 (767.7–1770.25)	844.5 (610.1–1244.4)	0.001	0.001	0.121	0.209
TNF-α (pg/mL)	37.7 (17.9–109.5)	192.8 (120.4–819.4)	133.5 (55.45–606.95)	<0.001	<0.001	0.01	0.579

* chi-square test,

** Student’s t-test,

T Mann-Whitney U test, for other variables, the Kruskal-Wallis test were applied.

DAS-28: Disease Activity Score-28, ESR: Erythrocyte Sedimentation Rate, CRP: C-Reactive Protein, RF: Rheumatoid Factor, anti-CCP: Anti-Cyclic Citrullinated Peptide, IL-40: Interleukin-40, IL-4: Interleukin-4, TGF-β1: Transforming Growth Factor Beta 1, TNF-α: Tumor Necrosis Factor Alpha.

**Table 2:** Comparison of ELISA parameters between dual-seropositive and dual-seronegative groups for naive RA patients.

Parameter	Dual positive (n = 12)	Dual negative (n = 10)	p-value
IL-40 (pg/mL)	287.2 ± 131.2	334.1 ± 136.4	0.422
IL-4 (pg/mL)	14.6 ± 7	7 ± 2.4	0.004
TNF-α (pg/mL)	182.7 (65.6–1144.95)	193.7 (151.7–819.4)	0.487*
TGF-β1 (pg/mL)	1491.6 ± 674.8	1020.5 ± 545.4	0.087

* Mann-Whitney U test, and for other variables, Student’s t-test were applied.

Dual-seropositive (RF ≥ 14 IU/mL and anti-CCP ≥ 5 U/mL and dual-seronegative (RF < 14 IU/mL] and anti-CCP < 5 IU/mL]). IL-40: Interleukin-40, IL-4: Interleukin-4, TGF-β1: Transforming Growth Factor Beta 1, TNF-α: Tumor Necrosis Factor Alpha.
